# Hypoxic human proximal tubular epithelial cells undergo ferroptosis and elicit an NLRP3 inflammasome response in CD1c^+^ dendritic cells

**DOI:** 10.1038/s41419-022-05191-z

**Published:** 2022-08-27

**Authors:** Kurt T. K. Giuliani, Anca Grivei, Purba Nag, Xiangju Wang, Melissa Rist, Katrina Kildey, Becker Law, Monica S. Ng, Ray Wilkinson, Jacobus Ungerer, Josephine M. Forbes, Helen Healy, Andrew J. Kassianos

**Affiliations:** 1 Conjoint Internal Medicine Laboratory, Chemical Pathology, Pathology Queensland, Brisbane, QLD Australia; 2 Kidney Health Service, Royal Brisbane and Women’s Hospital, Brisbane, QLD Australia; 3 Faculty of Medicine, University of Queensland, Brisbane, QLD Australia; 4 Institute of Health and Biomedical Innovation/School of Biomedical Sciences, Queensland University of Technology, Brisbane, QLD Australia; 5 Institute of Molecular Biosciences, University of Queensland, Brisbane, QLD Australia; 6 Department of Nephrology, Princess Alexandra Hospital, Brisbane, QLD Australia; 7 Mater Research Institute, University of Queensland, Brisbane, QLD Australia

**Keywords:** End-stage renal disease, Cell death, Cell death and immune response

## Abstract

Inflammasomes are multiprotein platforms responsible for the release of pro-inflammatory cytokines interleukin (IL)-1β and IL-18. Mouse studies have identified inflammasome activation within dendritic cells (DC) as pivotal for driving tubulointerstitial fibrosis and inflammation, the hallmarks of chronic kidney disease (CKD). However, translation of this work to human CKD remains limited. Here, we examined the complex tubular cell death pathways mediating inflammasome activation in human kidney DC and, thus, CKD progression. Ex vivo patient-derived proximal tubular epithelial cells (PTEC) cultured under hypoxic (1% O_2_) conditions modelling the CKD microenvironment showed characteristics of ferroptotic cell death, including mitochondrial dysfunction, reductions in the lipid repair enzyme glutathione peroxidase 4 (GPX4) and increases in lipid peroxidation by-product 4-hydroxynonenal (4-HNE) compared with normoxic PTEC. The addition of ferroptosis inhibitor, ferrostatin-1, significantly reduced hypoxic PTEC death. Human CD1c^+^ DC activated in the presence of hypoxic PTEC displayed significantly increased production of inflammasome-dependent cytokines IL-1β and IL-18. Treatment of co-cultures with VX-765 (caspase-1/4 inhibitor) and MCC950 (NLRP3 inflammasome inhibitor) significantly attenuated IL-1β/IL-18 levels, supporting an NLRP3 inflammasome-dependent DC response. In line with these in vitro findings, in situ immunolabelling of human fibrotic kidney tissue revealed a significant accumulation of tubulointerstitial CD1c^+^ DC containing active inflammasome (ASC) specks adjacent to ferroptotic PTEC. These data establish ferroptosis as the primary pattern of PTEC necrosis under the hypoxic conditions of CKD. Moreover, this study identifies NLRP3 inflammasome signalling driven by complex tubulointerstitial PTEC-DC interactions as a key checkpoint for therapeutic targeting in human CKD.

## Introduction

Chronic kidney disease (CKD) is a rapidly increasing healthcare burden, affecting ~275 million people globally [1]. Irrespective of its aetiology or its associated co-morbidities, the key pathobiological features of CKD are inflammation and fibrosis within the tubulointerstitial compartment [2, 3]. The structural and pathophysiological changes that arise from this tubulointerstitial damage generate a localised hypoxic environment [4, 5]. Prolonged hypoxia triggers additional pathobiology that includes tubular cell death/necrosis, resulting in the release of damage-associated molecular patterns (DAMP). Tubulointerstitial immune cells, such as dendritic cells (DC), detect these DAMP via a suite of pattern recognition receptors, including nucleotide-binding oligomerization domain (NOD)-like receptors (NLR) [6], becoming activated and driving pro-inflammatory processes that culminate in ongoing tubulointerstitial fibrosis [7, 8]. Despite the high prevalence of CKD, the specific tubular cell death-immune cell interactions that drive this hypoxic-mediated inflammatory loop remain poorly defined in humans.

Proximal tubular epithelial cells (PTEC) of the kidney are central players in CKD [9]. PTEC energy is primarily generated through mitochondrial fatty acid oxidation, an oxygen-intensive process [10, 11]. Thus, PTEC are particularly susceptible to hypoxic-driven mitochondrial dysfunction and cell death in CKD [12]. Studies have previously shown that hypoxic PTEC, in concert with pro-inflammatory factors (e.g., interleukin (IL)-18), significantly enhance pathogenic immune cell activation and cytotoxic licensing [13]. Supporting this concept, elevated levels of IL-1β and IL-18 are reported in human fibrotic kidney tissue [13]. Both cytokines are members of the IL-1 family of pro-inflammatory cytokines [14, 15]. However, the functional contribution of hypoxic PTEC death in driving IL-1β/IL-18 release within the diseased tubulointerstitium and the immunological source of these pro-inflammatory cytokines in human CKD remain elusive.

The secretion of active IL-1β and IL-18 is controlled by inflammasomes, cytosolic multiprotein response and signalling platforms [16–18]. The NLRP3 (nucleotide-binding domain and leucine-rich repeat-containing family, pyrin domain-containing 3) inflammasome is the best characterised of the inflammasomes [19]. Canonical NLRP3 inflammasome activation by DAMP signals involves both priming and activation steps that drive the formation of the inflammasome-caspase-1 holoenzyme. The formation of the inflammasome-caspase-1 complex is mediated by the adaptor protein ASC (apoptosis-associated speck-like protein containing a caspase activation and recruitment domain), which facilitates the recruitment and autolytic cleavage of caspase-1 on the inflammasome platform [19, 20]. Once cleaved, the active caspase-1 drives the proteolytic maturation and subsequent release of active IL-1β and IL-18 [20].

Experimental murine models of CKD have identified NLRP3 inflammasome activation as pivotal in promoting disease progression [21–23]. In particular, mouse dendritic cells (DC) within the diseased tubulointerstitium respond via the NLRP3-ASC-caspase-1 axis and are proposed to be the predominant source of active IL-1β [21, 24, 25]. In humans, the NLRP3 inflammasome has been strongly associated with tubulointerstitial injury/fibrosis and CKD progression [22, 26–30]. We have previously shown significantly increased numbers of activated human CD1c^+^ conventional DC type 2 (cDC2) in fibrotic kidney tissue [9]. These DC are localised to the tubulointerstitial compartment [9] and, thus, are well positioned to sense DAMP released by necrotic PTEC within the hypoxic CKD microenvironment.

In this study, we demonstrate that patient-derived primary PTEC cultured under hypoxic conditions modelling the human CKD microenvironment: (1) exhibit pathobiological features of ferroptotic cell death; and (2) trigger NLRP3 inflammasome-dependent IL-1β/IL-18 secretion in CD1c^+^ DC. Moreover, we confirm these pathogenic cell-cell interactions in human fibrotic kidneys, identifying a significant accumulation of tubulointerstitial CD1c^+^ DC containing active inflammasome (ASC) specks adjacent to ferroptotic PTEC. Our data establish NLRP3 inflammasome signalling driven by complex tubulointerstitial PTEC-CD1c^+^ DC interactions as a novel clinical target for therapeutic translation in human CKD.

## Results

### Human primary PTEC display mitochondrial dysfunction and DNA damage under hypoxic conditions

**Fig. 1:**
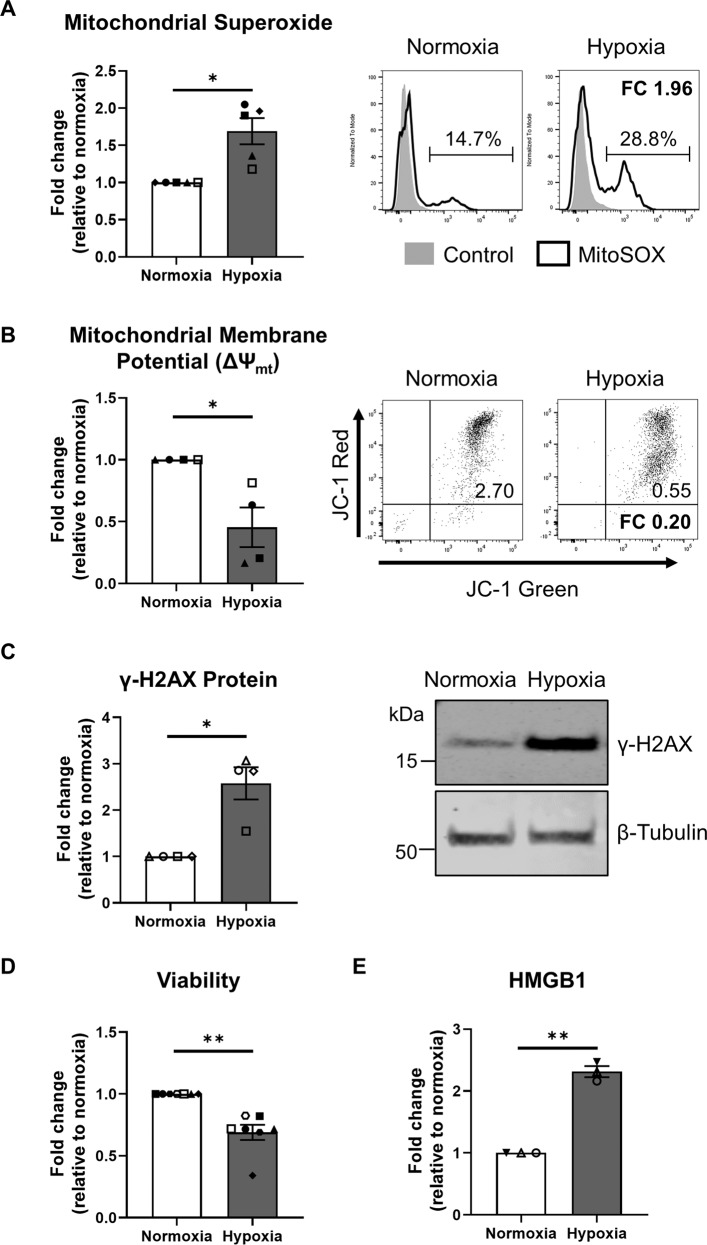
Hypoxia induces pathways of mitochondrial oxidative damage/dysfunction and DNA damage in human primary PTEC. **A** Left panel: fold changes (relative to normoxia) in mitochondrial superoxide levels (% MitoSOX^+^ cells) for PTEC cultured under normoxic and hypoxic conditions. Bar graphs represent mean ± standard error mean (SEM). Symbols represent individual donor PTEC; *n* = 5. *P < 0.05, paired *t* test. Right panel: representative MitoSOX staining (black unfilled) compared with unstained control (grey filled) for PTEC cultured under normoxic and hypoxic conditions. Mitochondrial superoxide levels (% MitoSOX^+^ cells) are presented for each histogram, with fold change (FC) value relative to normoxic PTEC also shown. **B** Left panel: Fold changes (relative to normoxia) in mitochondrial membrane potential [measured as ratio of ΔMFI JC-1 red/ΔMFI JC-1 green; with delta median fluorescence intensity (ΔMFI) representing MFI test – MFI unstained control] for PTEC cultured under normoxic and hypoxic conditions. Bar graphs represent mean ± SEM. Symbols represent individual donor PTEC; *n* = 4. *P < 0.05, paired *t* test. Right panel: representative JC-1 dot plots of PTEC cultured under normoxic and hypoxic conditions. Mitochondrial membrane potential (ΔΨ_mt_) values are presented for each histogram, with fold change (FC) value relative to normoxic PTEC also shown. **C** Left panel: fold changes (relative to normoxia) in γ-H2AX protein levels (as a ratio of loading control β-tubulin) for PTEC cultured under normoxic and hypoxic conditions. Bar graphs represent mean ± SEM. Symbols represent individual donor PTEC; *n* = 4. *P < 0.05, paired *t* test. Right panel: western blot for γ-H2AX for PTEC cultured under normoxic and hypoxic conditions (15 µg total protein per lane). Representative images from one of four donor PTEC are presented; full and uncropped western blot available as Supplementary Material. **D**, **E** Fold changes (relative to normoxia) in cell viability (MTT assay) (**D**) and HMGB1 (ELISA) (**E**) for PTEC cultured under normoxic and hypoxic conditions. Bar graphs represent mean ± SEM. Symbols represent individual donor PTEC; *n* = 7 for viability data and *n* = 3 for HMGB1 data. **P < 0.01, paired *t* test.

We have previously established a pathogenic function for human primary PTEC under conditions of hypoxia, an established driver of CKD [13, 31]. We extend on these previous reports to examine, for the first time, the pathobiological profile of human primary PTEC cultured under hypoxic (1% O_2_) versus normoxic control (21% O_2_) conditions. Hypoxic PTEC displayed significantly elevated mitochondrial superoxide (O_2_∙^−^) production (Fig. 1A), significantly reduced mitochondrial function (↓ mitochondrial membrane potential; ΔΨ_mt_) (Fig. 1B), significantly increased expression of the DNA damage marker γ-H2AX (Fig. 1C) and significantly reduced cell viability (Fig. 1D) compared with normoxic conditions. Examination of hypoxic PTEC culture supernatants showed significantly elevated levels of high mobility group box protein-1 (HMGB1) (Fig. 1E), a pro-inflammatory DAMP released after necrotic cell damage [32].

### Human primary PTEC undergo ferroptotic cell death under hypoxic conditions

**Fig. 2:**
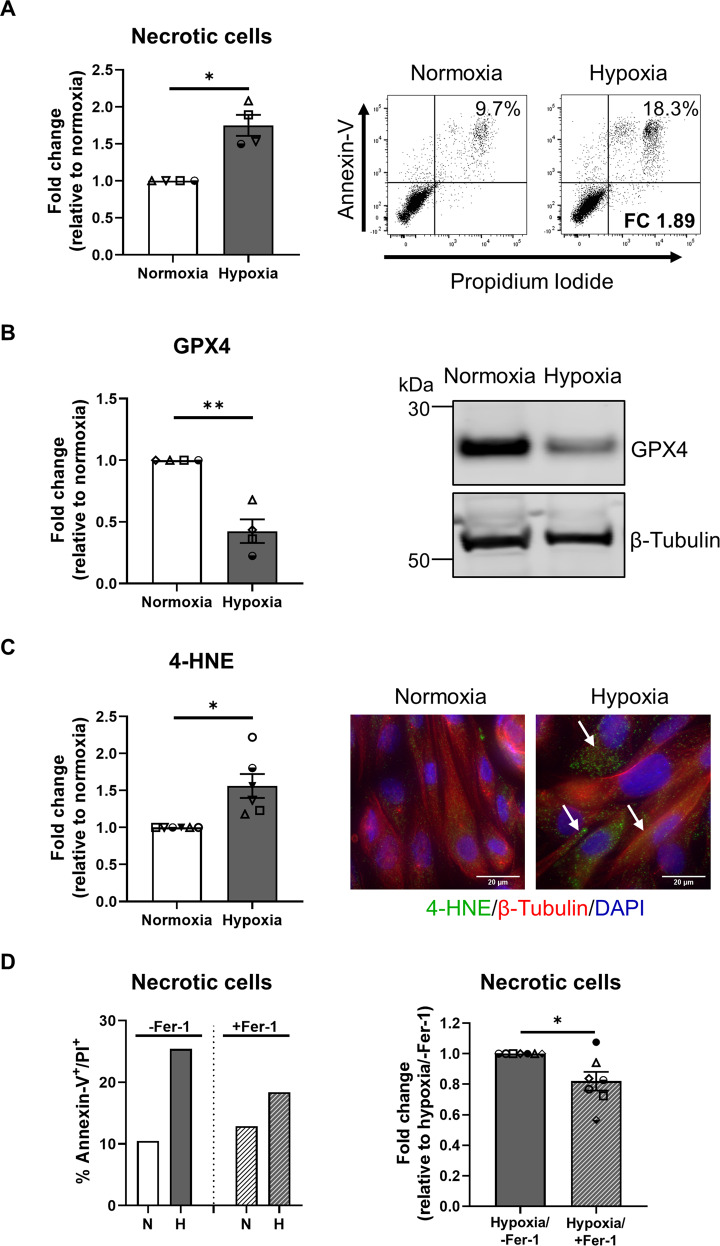
Hypoxia induces ferroptosis in human primary PTEC. **A** Left panel: fold changes (relative to normoxia) in cellular necrosis (% Annexin-V^+^ PI^+^ cells) for PTEC cultured under normoxic and hypoxic conditions. Bar graphs represent mean ± SEM. Symbols represent individual donor PTEC; *n* = 4. *P < 0.05, paired *t* test. Right panel: representative donor Annexin-V/PI dot plots of PTEC cultured under normoxic and hypoxic conditions. The percentage of Annexin-V^+^ PI^+^ necrotic cells for each dot plot are presented, with fold change (FC) value relative to normoxic PTEC also shown. **B** Left panel: fold changes (relative to normoxia) in GPX4 protein levels (as a ratio of loading control β-tubulin) for PTEC cultured under normoxic and hypoxic conditions. Bar graphs represent mean ± SEM. Symbols represent individual donor PTEC; *n* = 4. **P < 0.01, paired *t* test. Right panel: GPX4 western blot for PTEC cultured under normoxic and hypoxic conditions (15 µg total protein per lane). Representative images from one of four donor PTEC are presented; full and uncropped western blot available as Supplementary Material. **C** Left panel: fold changes (relative to normoxia) in 4-HNE levels (measured as mean corrected total cellular fluorescence (CTCF) of >60 cells per condition) for PTEC cultured under normoxic and hypoxic conditions. Bar graphs represent mean ± SEM. Symbols represent individual donor PTEC; *n* = 6. *P < 0.05, paired *t* test. Right panel: immunofluorescent labelling of representative PTEC cultured under normoxic and hypoxic conditions and stained for 4-HNE (green), β-tubulin (red) and DAPI (blue). 4-HNE positivity is highlighted with white arrows. Scale bars represent 20 µm. **D** Left panel: representative donor bar graph of cellular necrosis for PTEC cultured under normoxic (N) and hypoxic (H) conditions in the absence (-Fer-1; DMSO vehicle control) or presence of ferrostatin-1 (+Fer-1). Right panel: fold changes (relative to hypoxia/-Fer-1) in cellular necrosis (% Annexin-V^+^ PI^+^ cells) for hypoxic PTEC cultured in the absence or presence of ferrostatin-1. Bar graphs represent mean ± SEM. Symbols represent individual donor PTEC; *n* = 7. *P < 0.05, paired *t* test.

Further interrogation revealed significantly increased cellular necrosis (% Annexin-V^+^ propidium iodide (PI)^+^ cells) in hypoxic PTEC compared with normoxic PTEC (Fig. 2A). The discrete molecular pathways of cell death were next assessed. Expression levels of apoptosis marker cleaved caspase-3 (Supplementary Fig. S2A) and mitochondrial permeability transition (MPT)-regulated necrosis marker peptidyl-prolyl isomerase F (PPIF) (Supplementary Fig. S2A) were comparable between hypoxic and normoxic PTEC, whilst the necroptosis marker phosphorylated mixed lineage kinase domain-like protein (pMLKL) was not detectable in either PTEC population (Supplementary Fig. S2B). In contrast, significantly decreased expression of lipid repair enzyme glutathione peroxidase 4 (GPX4) (Fig. 2B and Supplementary Fig. S2C), and significantly increased lipid peroxidation (Supplementary Fig. S2D), and lipid peroxidation by-product 4-hydroxynonenal (4-HNE) (Fig. 2C) were detected in hypoxic PTEC compared with normoxic PTEC. Decreased GPX4 and increased 4-HNE are both hallmarks of ferroptotic cell death, a form of regulated necrosis characterised by the accumulation of iron-dependent lipid peroxides [33]. Of note, the addition of established ferroptosis/lipid peroxidation inhibitor ferrostatin-1 significantly attenuated cellular necrosis in hypoxic PTEC (Fig. 2D). These data establish ferroptosis as a key form of human PTEC death under hypoxic conditions.

### Co-localisation of ferroptotic PTEC with CD1c^+^ DC in fibrotic kidney tissue

**Fig. 3:**
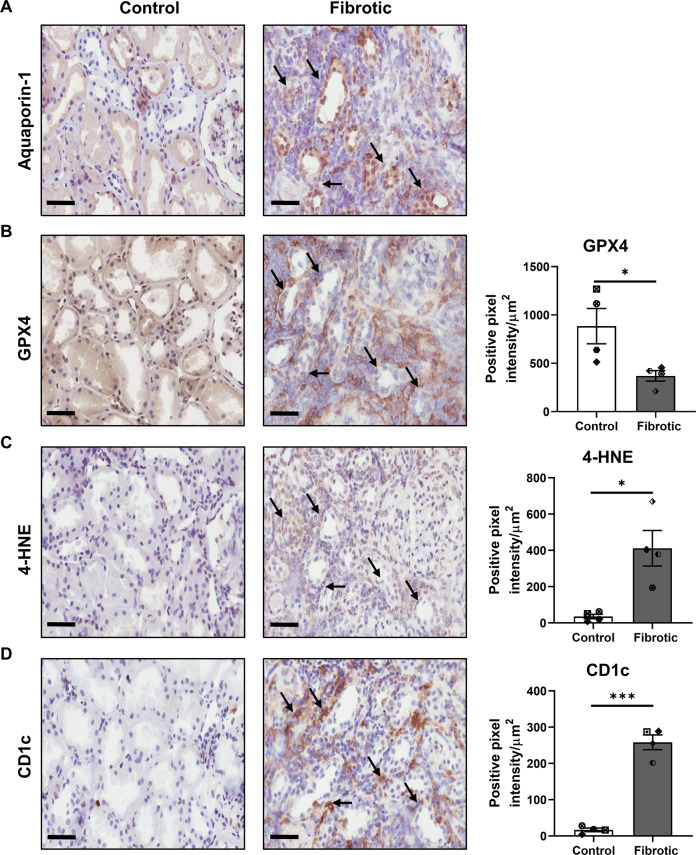
Co-localisation of human CD1c^+^ DC with ferroptotic PTEC in fibrotic kidney tissue. Representative serial section immunohistochemical (IHC) staining of control/non-fibrotic (left panel) and fibrotic (middle panel) kidney tissue probed for (**A**) aquaporin-1, (**B**) GPX4, (**C**) 4-HNE and (**D**) CD1c. Regions of CD1c^+^ DC co-localisation with aqupaporin-1^+^ PTEC displaying evidence of ferroptotic cell death (↓ GPX4 and ↑ 4-HNE) are highlighted with black arrows. Scale bars represent 60 µm. Quantitative analysis (positive pixel intensity/µm^2^ total area) of IHC staining in control and fibrotic tissue is presented (right panels). Symbols represent values for individual donor tissue. Results represent mean ± SEM of values from four randomly selected areas for each tissue sample. *P < 0.05, ***P < 0.001, Welch’s *t* test.

To translate these in vitro data to an in situ model, we performed immunohistochemical (IHC) staining of human fibrotic kidney tissue. PTEC in control/non-fibrotic and fibrotic kidney tissue were identified based on the expression of aquaporin-1 (AQP-1) (Fig. 3A), a marker restricted to the proximal regions of the tubular compartment [34]. Serial section analysis showed a selective reduction in GPX4 levels and increased 4-HNE in AQP-1^+^ PTEC within fibrotic kidney tissue (highlighted with black arrows) compared with control samples (Fig. 3B, C), supporting the concept of ferroptotic PTEC death in tubulointerstitial fibrosis. Quantitative analysis confirmed these findings, with significantly decreased GPX4 expression and significantly elevated 4-HNE in fibrotic tissue compared with control tissue (Fig. 3B, C).

Moreover, we showed a significantly increased tubulointerstitial accumulation of CD1c^+^ cells (with dendritic morphology) in fibrotic kidney tissue (Fig. 3D). Notably, these CD1c^+^ DC were both localised to sites of tubulointerstitial injury and adjacent to ferroptotic PTEC (Fig. 3D). These collective findings provide evidence of spatial co-localisation of ferroptotic PTEC with CD1c^+^ DC in tubulointerstitial fibrosis.

### CD1c^+^ DC activated in the presence of hypoxic PTEC produce significantly increased levels of inflammasome cytokines IL-1β/IL-18

We established an in vitro co-culture model to examine the mechanistic interactions which could occur between adjacent PTEC and CD1c^+^ DC in fibrotic kidneys. We isolated human CD1c^+^ DC by immuno-magnetic selection/flow cytometry sorting (>99% purity) and examined their pro-inflammatory cytokine profiles following co-culture with pre-conditioned normoxic (21% O_2_) or hypoxic (1% O_2_) PTEC in the absence or presence of poly I:C, a double-stranded RNA (dsRNA) analogue and TLR3 agonist. Poly I:C is a physiologically relevant activator, with TLR3 reported to be a critical sensor of endogenous RNA released during tissue necrosis [35, 36].

**Fig. 4:**
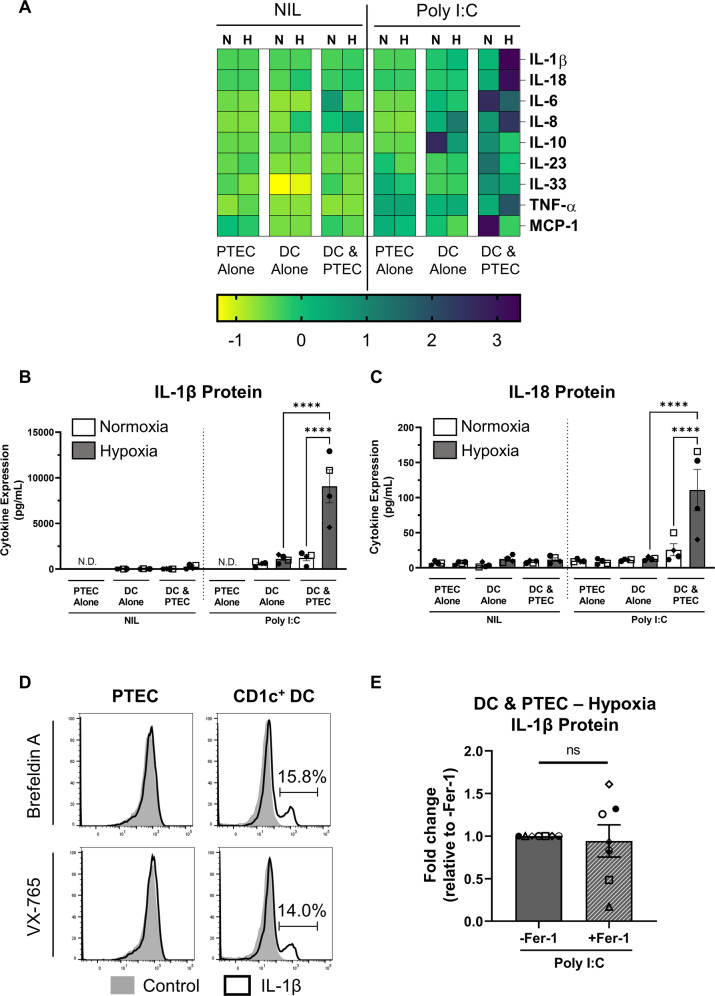
CD1c^+^ DC activated in the presence of hypoxic PTEC secrete significantly increased levels of inflammasome cytokines IL-1β/IL-18. **A** Heatmap representing secreted cytokine levels following 24-h co-culture (DC & PTEC) of flow cytometry sorted CD1c^+^ DC with pre-conditioned normoxic (N; 21% O_2_) or hypoxic (H; 1% O_2_) PTEC in the absence (Nil) or presence of poly I:C. PTEC alone and DC alone cultures are included as controls; *n* = 4. The colour bar represents the z-score. Yellow indicates low expression; purple indicates high expression. IL-12p70, IL-17, interferon (IFN)-α and IFN-γ were not detectable in any culture conditions (data not shown). **B**, **C** Secreted IL-1β (**B**) and IL-18 (**C**) protein levels (measured by LEGENDplex™ assay; pg/ml) following 24-h co-culture (DC & PTEC) of flow cytometry sorted CD1c^+^ DC with pre-conditioned normoxic (white bars) or hypoxic (grey bars) PTEC in the absence (Nil) or presence of poly I:C. PTEC alone and DC alone cultures are included as controls; N.D. not detectable. Bar graphs represent mean ± SEM. Symbols represent individual donor PTEC experiments; *n* = 4. ****P < 0.0001, one-way ANOVA with Tukey’s multiple-comparison test. **D** Flow cytometric detection of intracellular IL-1β protein following 4-h co-culture of magnetic bead-enriched CD1c^+^ DC with pre-conditioned hypoxic PTEC in the presence of poly I:C. Co-cultures were treated prior to the 4-h culture period with either brefeldin A or VX-765 to enable intracellular IL-1β accumulation/detection. Representative flow cytometric histograms of IL-1β staining (black unfilled) compared with unstained control (grey filled) for PTEC (gated on live, single, CD45^−^ cells) and CD1c^+^ DC (gated on live, single, CD45^+^ lineage^−^ HLA-DR^+^ CD1c^+^ cells) are presented. One representative of three individual donor PTEC experiments is shown. **E** Fold changes (relative to –Fer-1) in secreted IL-1β levels (measured by ELISA; pg/ml) following 24-h co-culture (DC & PTEC) of magnetic bead-enriched CD1c^+^ DC with pre-conditioned hypoxic PTEC and poly I:C in the absence (-Fer-1) or presence of ferrostatin-1 (+Fer-1). Bar graphs represent mean ± SEM. Symbols represent individual donor PTEC experiments; *n* = 7. ns not significant, paired *t* test.

In the absence of poly I:C stimulation, no to minimal changes in soluble cytokine levels were detected by LEGENDplex™ assay in the supernatants of either normoxic or hypoxic PTEC-DC co-cultures (or PTEC/DC alone control cultures) (Fig. 4A–C and Supplementary Fig. S3). In response to poly I:C, IL-6 was significantly increased in both normoxic and hypoxic PTEC-DC co-cultures compared with their equivalent DC alone control cultures, whilst significantly elevated levels of monocyte chemoattractant protein-1 (MCP-1) and IL-8 were detected in normoxic and hypoxic co-cultures, respectively (Fig. 4A and Supplementary Fig. S3). Of particular note, significantly increased levels of inflammasome-dependent cytokines IL-1β and IL-18 were detected in hypoxic PTEC-DC co-culture supernatants compared with both normoxic co-cultures and hypoxic DC alone (Fig. 4A–C).

To enable intracellular localisation/detection of IL-1β, hypoxic PTEC-DC co-cultures (in the presence of poly I:C) were pre-treated with either protein transport inhibitor brefeldin A to block endoplasmic reticulum/golgi complex transport of inflammasome components or caspase-1/4 inhibitor VX-765. Both modes of inhibition showed IL-1β expression in hypoxic co-cultures restricted to CD1c^+^ DC (~15%), with no detectable levels in PTEC (Fig. 4D). These data point to CD1c^+^ DC as a novel source of IL-1β within the hypoxic microenvironment of human CKD.

The addition of ferrostatin-1 to inhibit hypoxic PTEC ferroptosis elicited a partial attenuation of this CD1c^+^ DC-derived IL-1β, with reduced cytokine levels in co-culture supernatants in 4/7 donor PTEC experiments (Fig. 4E). We conclude that although PTEC ferroptosis plays a role in CD1c^+^ DC IL-1β production, there are other, dominant pathways (i.e., inflammasome signalling) in the complex and multifactorial PTEC-DC crosstalk of human CKD.

### Hypoxic PTEC trigger CD1c^+^ DC-derived IL-1β/IL-18 secretion via NLRP3 inflammasome activation

**Fig. 5:**
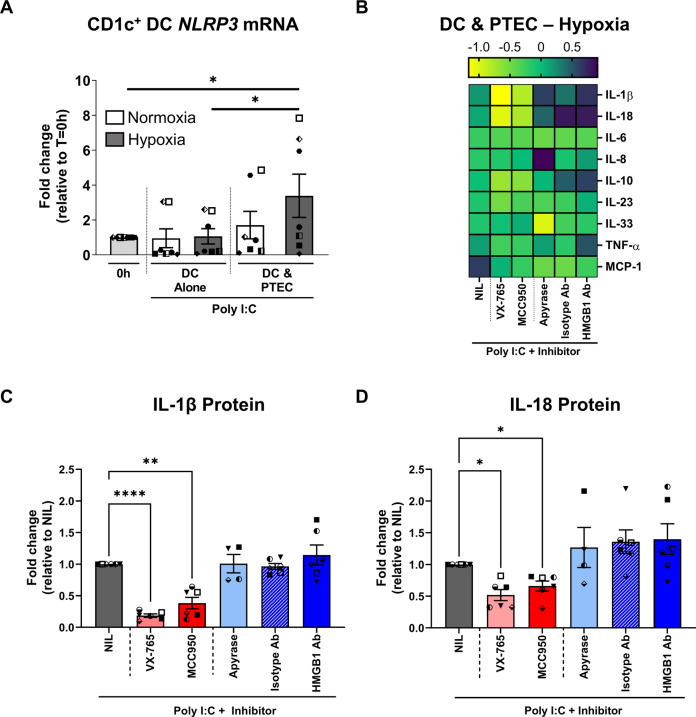
Hypoxic PTEC trigger CD1c^+^ DC-derived IL-1β/IL-18 via activation of the NLRP3 inflammasome. **A** *NLRP3* mRNA expression relative to housekeeping gene β-2-microglobulin (*B2M)* in flow cytometry sorted CD1c^+^ DC freshly isolated (0 h) or following 24-h co-culture (DC & PTEC) with pre-conditioned normoxic (white bar) or hypoxic (grey bar) PTEC in the presence of poly I:C. DC alone cultures are included as controls. Bar graphs represent mean ± SEM. Symbols represent individual donor PTEC experiments; *n* = 7. *P < 0.05, one-way ANOVA with Tukey’s multiple-comparison test. **B** Heatmap representing secreted cytokine levels following 24-h co-culture (DC & PTEC) of magnetic bead-enriched CD1c^+^ DC with pre-conditioned hypoxic PTEC and poly I:C in the absence (NIL) or presence of inflammasome inhibitors (VX-765, MCC950) or DAMP inhibitors (apyrase, isotype control or HMGB1 antibody); *n* = 6. The colour bar represents the z-score. Yellow indicates low expression; purple indicates high expression. **C**, **D** Fold changes (relative to NIL inhibitor) in secreted IL-1β (**C**) and IL-18 (**D**) protein levels (measured by LEGENDplex™ assay) following 24-h co-culture (DC & PTEC) of magnetic bead-enriched CD1c^+^ DC with pre-conditioned hypoxic PTEC and poly I:C in the absence (NIL) or presence of inflammasome inhibitors (VX-765, MCC950) or DAMP inhibitors (apyrase, isotype control or HMGB1 antibody). Bar graphs represent mean ± SEM. Symbols represent individual donor PTEC experiments; *n* = 6. *P < 0.05; **P < 0.01; ****P < 0.0001, one-way ANOVA with Tukey’s multiple-comparison test.

Mechanistic investigations were undertaken to confirm the functional role of inflammasome-dependent signalling, in particular, NLRP3 inflammasome activation, in this human CKD model. The expression of CD1c^+^ DC *NLRP3* mRNA was evaluated in poly I:C-stimulated cultures. Poly I:C stimulation of normoxic or hypoxic DC alone failed to induce *NLRP3* mRNA expression (Fig. 5A). This is in line with previous studies establishing extracellular poly I:C as a weak activator of the NLRP3 inflammasome [37, 38]. In contrast, significantly increased *NLRP3* mRNA was detected in CD1c^+^ DC from hypoxic co-cultures (Fig. 5A). These data suggest that hypoxic PTEC-derived DAMP trigger CD1c^+^ DC via activation of the NLRP3 inflammasome.

Neutralisation studies were performed to further evaluate these hypoxic PTEC-DC interactions. Established inhibitors for caspase-1/4 (VX-765) and the NLRP3 inflammasome (MCC950) [39, 40] significantly attenuated IL-1β (Fig. 5B, C) and IL-18 levels (Fig. 5B, D) in poly I:C-stimulated hypoxic co-culture supernatants, confirming the NLRP3 inflammasome as the predominant inflammasome in this human CKD model. Upstream targeting of inflammasome-activating DAMP using a HMGB1 neutralising antibody or apyrase (extracellular adenosine triphosphate (ATP) inhibitor) did not significantly modulate IL-1β (Fig. 5B, C) or IL-18 (Fig. 5B, D). No significant differences were observed for cytokines other than IL-1β or IL-18 in the presence of inflammasome or DAMP inhibitors (Fig. 5B).

### Significantly elevated tubulointerstitial CD1c^+^ DC with ASC specks in fibrotic kidneys

**Fig. 6:**
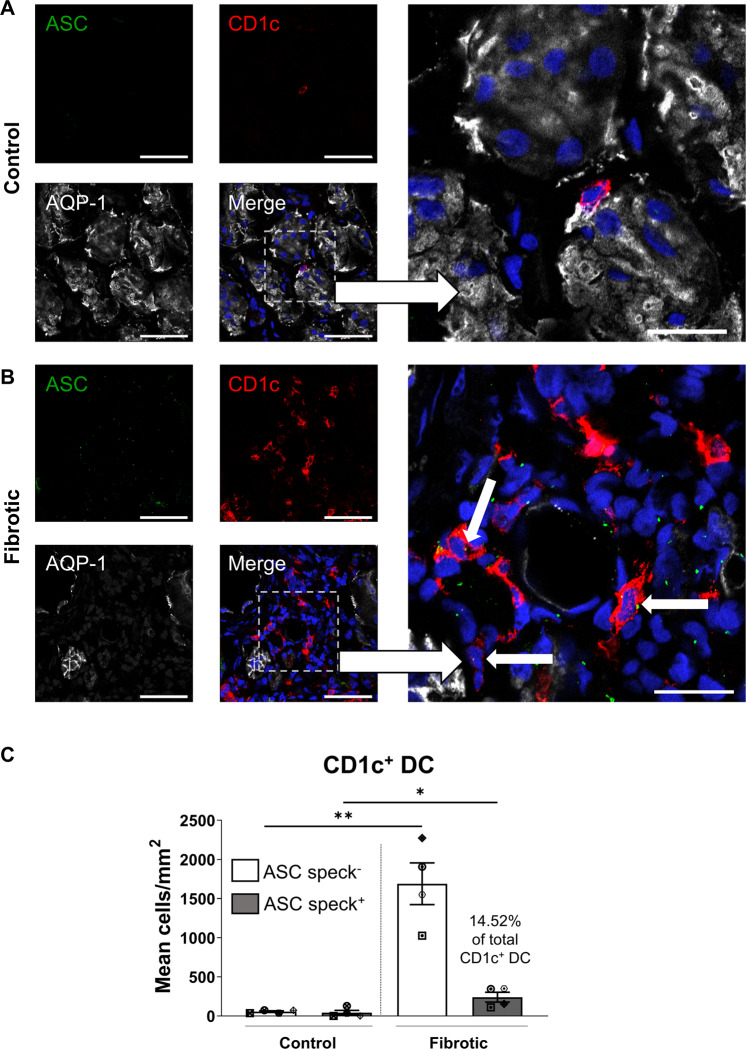
Significantly elevated tubulointerstitial CD1c^+^ DC with ASC specks in fibrotic kidney tissue. **A**, **B** Immunofluorescent labelling of frozen sections from control/non-fibrotic (**A**) and fibrotic kidney tissue (**B**) stained for ASC (green), CD1c (red), aquaporin-1 (AQP-1) (white) and DAPI (blue). Scale bars represent 50 µm for small frames (left panels) and 20 µm for large frames (right panels). ASC-speck-positive CD1c^+^ DC are highlighted with white arrows. **C** Quantification (mean cells/mm^2^) of ASC-speck-negative CD1c^+^ DC (white bars) and ASC-speck-positive CD1c^+^ DC (grey bars) in control/non-fibrotic kidney tissue (*n* = 4) and fibrotic kidney tissue (*n* = 4). Symbols represent values for individual donor tissue. Results represent mean ± SEM of values from five randomly selected areas for each tissue sample. *P < 0.05, **P < 0.01, Welch’s *t* test.

A hallmark of NLRP3 inflammasome activation is the ASC speck, a cytosolic supramolecular aggregate of the inflammasome adaptor protein ASC [41]. We demonstrated, for the first time, tubulointerstitial CD1c^+^ DC with punctate ASC specks in fibrotic kidney tissue. These ASC-speck-positive CD1c^+^ DC were positioned in close proximity to AQP-1^+^ PTEC (Fig. 6A, B). Quantitative analysis confirmed a significantly increased accumulation of ASC-speck-positive CD1c^+^ DC in fibrotic kidney tissue compared with control/non-fibrotic tissue (Fig. 6C), with 14.52 ± 8.18% of CD1c^+^ DC containing ASC specks. Of note, this DC population was identified as a key cellular source of ASC specks in fibrotic kidneys, with 31.61 ± 16.23% of total ASC-speck-positive cells identified as CD1c^+^ DC. Collectively, these findings support a specialised pathobiological role for PTEC-DC crosstalk in triggering inflammasome activation in human CKD.

## Discussion

Pro-inflammatory cytokines IL-1β and IL-18 are potent molecules in the propagation of tubulointerstitial hypoxia/fibrosis, the pathological hallmarks of CKD [13, 42]. Here, we present data identifying CD1c^+^ DC as a key source of IL-1β/IL-18 in human CKD, mediated via complex NLRP3 inflammasome-dependent interactions with damaged PTEC within the hypoxic microenvironment.

We established an in vitro model of tubulointerstitial hypoxia to dissect the mechanistic interplay between co-localised PTEC and CD1c^+^ DC in human CKD. Under hypoxic conditions, our human primary PTEC displayed evidence of mitochondrial dysfunction (↑ mitochondrial O_2_∙^−^, ↓ ΔΨ_mt_) leading to DNA damage (↑ γ-H2AX). The pathobiological link between mitochondrial oxidative stress and impaired mitochondrial function has been established in experimental CKD models and also kidney tissue from CKD patients [43–47]. Moreover, a recent study of kidney tissue from CKD patients reported extensive PTEC DNA damage, marked by phosphorylation of the histone H2A variant H2AX (γ-H2AX) [48]. The identification of these shared pathobiological pathways in our hypoxic human primary PTEC validates our in vitro model as a clinically relevant system for examining downstream tubulointerstitial disease processes (i.e., tubular cell death) observed in CKD patients.

If unresolved, sustained mitochondrial dysregulation triggers pathways of regulated cell death (necrosis). We demonstrated the selective induction of PTEC ferroptosis (↓ GPX4, ↑ 4-HNE) both in our in vitro hypoxic model of human CKD and in situ within fibrotic kidney tissue. Ferroptotic cell death is an iron- and reactive oxygen species (ROS)-dependent necrosis [33]. Ferroptotic tubular cell death has been reported in human kidney biopsy specimens of diverse aetiologies, including acute tubular injury patients [49, 50]. Our present study extends these findings to the hypoxic microenvironment of human CKD. Hypoxia can promote ferroptosis by damaging mitochondrial membranes, leading to elevated intracellular levels of (i) hydrogen peroxide (H_2_O_2_)—generated by dismutation of excess mitochondrial superoxide (O_2_∙^−^); and (ii) labile/free iron released from mitochondrial iron-containing proteins (e.g., cytochrome *c*) [51]. Subsequent iron-H_2_O_2_ interactions yield highly toxic free radicals—hydroxyl radicals (OH ∙ )—via the Fenton reaction [52]. These free radicals react with polyunsaturated fatty acids to generate lipid peroxides [53]. The lipid repair enzyme GPX4 can reduce these toxic lipid peroxides to their alcohol form [54]. A deficiency in GPX4 function/expression therefore results in an accumulation of lipid peroxides and their degradation products (i.e., oxidised phospholipids and 4-HNE) that, in turn, trigger ferroptotic cell death [55]. Notably, increased plasma levels of 4-HNE have been reported in CKD patients [56]. Here, we map this lipid peroxidation by-product to ferroptotic PTEC under in vitro hypoxic conditions and in human fibrotic kidney tissue.

Our findings substantiate the “wave of death” hypothesis in which intercellular propagation of ferroptotic cell death, and its release of pro-inflammatory DAMP, accounts for ongoing kidney tubular injury and loss of nephrons [57, 58]. In particular, Ide et al. recently reported that ‘damage associated’ PTEC display both a dysregulation in the glutathione/GPX4 anti-ferroptotic defence pathway and promote a pro-inflammatory milieu via cell-cell signalling to myeloid cells [59]. Our study identifies tubulointerstitial CD1c^+^ DC as the critical myeloid cell population in human CKD, co-localising with ferroptotic PTEC in fibrotic kidney tissue and sensing PTEC-derived DAMP via the NLRP3 inflammasome. ASC is a key adaptor protein with a pivotal role in NLRP3 inflammasome assembly and activation [19]. Following inflammasome activation, ASC assembles into a large protein complex or ‘speck’ [60]. ASC specks are released from inflammasome-activated cells and accumulate in extracellular bodily fluids in human chronic inflammatory diseases [41, 60, 61]. Of particular relevance, ASC in the urine of CKD patients positively correlates with the concentration of proteinuria, a biomarker of kidney injury [62]. Our study confirms tubulointerstitial CD1c^+^ DC as a key immunological source of ASC specks within human fibrotic kidney tissue.

Our in vitro interrogation of the danger signals mediating CD1c^+^ DC inflammasome activation highlights the complexity of this cell-cell interaction. Firstly, triggering of CD1c^+^ DC with TLR3 agonist poly I:C was required to elicit an NLRP3 inflammasome response in our CKD model. TLR3 has been recently implicated in kidney fibrosis and inflammation via activation of the transforming growth factor (TGF)-β/Smad/TLR3 signalling pathway [63]. TLR3 recognition of self dsRNA released from necrotic cells also induces sterile inflammation [35, 36], with poly I:C representative of these endogenous RNA DAMP in our in vitro model of tubulointerstitial hypoxia. Most notably, extracellular poly I:C is a weak to non-activator of the NLRP3 inflammasome [37, 38], and was thus selected as a stimulus in our CKD model to trigger DC without masking the effect of inflammasome-activating DAMP from hypoxic PTEC. In line with these previous reports, the addition of poly I:C alone failed to induce CD1c^+^ DC *NLRP3* mRNA expression in our system, but importantly, sensitised the DC to subsequent NLRP3 inflammasome triggering by hypoxic PTEC.

Cellular products of lipid peroxidation (i.e., oxidized phospholipids) have been identified as inflammasome-activating DAMP [64–66]. However, pre-treatment of hypoxic PTEC with ferroptosis/lipid peroxidation inhibitor ferrostatin-1 only partially attenuated downstream CD1c^+^ DC IL-1β production (in 4/7 co-culture experiments), despite significantly blocking necrotic cell death in these PTEC. We conclude that there are alternate hypoxic PTEC-derived DAMP in addition to those directly associated with the ferroptotic cell death pathway. The functional role of other established inflammasome-activating DAMP (i.e., HMGB1 and ATP) was also examined. Accumulating evidence implicates both HMGB1 and ATP signalling pathways in the development of tubulointerstitial fibrosis [67–70]. However, inhibition of HMGB1 and extracellular ATP did not significantly modulate NLRP3 inflammasome activation in CD1c^+^ DC. Collectively, these data are suggestive of redundancies in PTEC-derived DAMP signalling to CD1c^+^ DC, mediated by complex and highly diverse inflammasome-activating stimuli. The corollary is that therapeutics targeting this component of the NLRP3 inflammasome-activation pathway in CD1c^+^ DC are likely to be unsuccessful in CKD.

Targeting IL-1β/IL-18 signalling downstream of inflammasome activation has been assessed as an alternate strategy in pre-clinical and clinical studies of CKD. Of these two pro-inflammatory cytokines, IL-1β is currently the prime focus of immunotherapeutic strategies targeting the inflammasome. The IL-1 receptor (IL-1R) antagonist, anakinra, was shown to be effective in the treatment of gout flares in patients with advanced CKD [71]. However, the investigators reported no significant improvement in kidney function [71]. Similarly, in high-risk atherosclerosis patients with CKD, treatment with canakinumab, an IL-1β neutralising antibody, had no beneficial effect on kidney function, but significantly reduced major adverse cardiovascular events [72]. We propose that effective renoprotection requires blocking both IL-1β and IL-18 by targeting upstream of their activation (i.e., the NLRP3 inflammasome with MCC950). The efficacy of sulfonylurea-containing compound MCC950 was evaluated in a phase II clinical trial of rheumatoid arthritis—however, this clinical study was suspended due to off-target hepatotoxicity [73]. Attention has since focussed on the development of less toxic MCC950 analogues, with IZD-334 (NCT04086602) and inzomelid (NCT04015076) both in clinical trials for the treatment of cryopyrin-associated periodic syndrome (CAPS) [74]. Our study provides the proof of concept for repurposing of such MCC950-related compounds for the therapeutic targeting of CD1c^+^ DC in CKD and other inflammatory kidney diseases.

Collectively, these results provide the first comprehensive molecular and functional characterisation of inflammasome signalling under the hypoxic conditions of human CKD. We provide evidence of ferroptotic PTEC death, release of putative DAMP and triggering of CD1c^+^ DC via the NLRP3 inflammasome within the hypoxic/fibrotic tubulointerstitium. The broader application of these findings will enable the development of novel therapeutic approaches with greater specificity—i.e., clinical testing of next-generation inflammasome inhibitors, engineered to avoid off-target effects, but not yet trialled in kidney diseases.

## Materials and methods

### Kidney tissue specimens

Kidney cortical tissue was obtained with informed patient consent from the macroscopically healthy portion of tumour nephrectomies, following approval by the Royal Brisbane and Women’s Hospital Human Research Ethics Committee (2002/011 and 2006/072). Kidney tissue was immediately divided for: (1) isolation and culture of human primary PTEC; (2) freezing in Tissue-Tek OCT compound (Sakura, Torrance, CA, USA) for immunohistochemical (IHC)/immunofluorescence (IF) analysis and (3) fixation in formalin for morphological assessment of interstitial fibrosis/tubular atrophy by kidney histopathologists in a blinded manner. Kidney tissue specimens displaying ≥5% interstitial fibrosis were deemed fibrotic, based on the Banff 97 working classification of kidney pathology [75]. Tissue specimens were grouped into ‘control/non-fibrotic’ (*n* = 17; 4 female/13 male; mean age of 56.3 ± 11.5 years) or ‘fibrotic’ cortical tissue (*n* = 5; 2 female/3 male; mean age of 54.4 ± 9.7 years) based on this histopathological assessment of the non-tumour kidney parenchyma.

### Isolation and culture of human primary PTEC

Human primary PTEC were purified from kidney cortical tissue following the method of Glynne and Evans [76] and then cultured in Defined Medium (DM) as previously described [77]. Human primary PTEC isolated from ‘control/non-fibrotic’ cortical tissue were used for in vitro experiments. All PTEC were used in experiments at passage 4.

### Hypoxic treatment of human primary PTEC to mimic the CKD microenvironment

PTEC were cultured to 70–80% confluence in DM in 24-well flat-bottom plates (unless otherwise specified). To prevent further proliferation, PTEC were then irradiated with 30 Gy. PTEC were further cultured for 72 h (unless otherwise specified) in fresh DM (1 ml volume) for normoxic PTEC (21% O_2_) or, for hypoxic PTEC (1% O_2_), in hypoxia pre-conditioned fresh DM (1 ml volume) in an InvivO_2_ 1000 Hypoxia Workstation (Ruskinn, Laftec, Bayswater North, Victoria, Australia). The expression of hypoxia-inducible factor 1α (HIF-1α) by hypoxic PTEC alone was confirmed by western blotting as previously described [31]. For inhibitor studies, 10 µM ferrostatin-1 (Sigma-Aldrich, St Louis, MO, USA) or 0.02% dimethyl sulfoxide (DMSO) vehicle control were added to PTEC cultures for the 72-h treatment period.

For further characterisation of normoxic versus hypoxic PTEC, cell culture supernatant was collected for analysis of soluble factors (e.g., HMGB1), with PTEC harvested by trypsin treatment for downstream experiments. PTEC mitochondrial superoxide production, mitochondrial function and necrosis were assessed by flow cytometry, with cell acquisition performed on an LSR Fortessa (BD Biosciences, San Jose, CA, USA) and data analysed with FlowJo software (TreeStar, Ashland, OR, USA). Harvested PTEC were also examined for protein expression by western blotting (detailed information for these PTEC assays in Supplementary Methods).

### Cell viability measurements of human primary PTEC

Cell viability of normoxic versus hypoxic human primary PTEC was investigated using the colorimetric MTT (3-(4,5-dimethylthiazol-2-yl)-2,5-diphenyltetrazolium bromide) Cell Proliferation Assay kit (Molecular Probes, Eugene, OR, USA) (detailed method provided in Supplementary Methods).

### 4-HNE immunofluorescence staining of human primary PTEC

PTEC were seeded (100,000 cells/well in DM) onto sterile coverslips in 24-well flat-bottom plates to 70–80% confluence. PTEC were irradiated with 30 Gy and then cultured in fresh, conditioned DM under normoxic or hypoxic conditions for 72 h for downstream 4-HNE immunofluorescence staining (detailed method provided in Supplementary Methods).

### Image-iT^TM^ lipid peroxidation assay

PTEC were seeded (30,000 cells/well in DM) into 96-well, black/clear bottom plates to 70–80% confluence. PTEC were irradiated with 30 Gy and then cultured for 48 h in fresh, conditioned DM under normoxic or hypoxic conditions for downstream staining/imaging with BODIPY 581/591 C11 reagent (Image-iT^TM^ lipid peroxidation kit, ThermoFisher Scientific) (detailed method provided in Supplementary Methods).

### PTEC-CD1c^+^ DC co-cultures

PTEC for co-culture experiments were seeded in DM in 96-well flat-bottom plates and grown to 70–80% confluence, irradiated with 30 Gy and then cultured for 72 h in 200 µl fresh, conditioned DM under normoxic or hypoxic conditions.

Human CD1c^+^ DC were purified from mononuclear cells using the Human CD1c (BDCA-1)^+^ Dendritic Cell Isolation kit (Mitenyi Biotec, Bergisch Gladbach, Germany). For selected experiments, CD1c^+^ DC were further purified by fluorescence-activated cell sorting (FACS) (additional DC isolation methodology in Supplementary Methods).

CD1c^+^ DC were resuspended at 1.33 × 10^6^ cells/ml in Complete Medium (CM) (components detailed in Supplementary Methods). CD1c^+^ DC (100,000 cells in CM; 75 µl total volume) were added to the pre-conditioned PTEC (without removal of PTEC DM culture medium) and co-cultured for 24 h (unless otherwise specified) under normoxic or hypoxic conditions (10% foetal bovine serum (FBS) final concentration; 275 µL final volume). PTEC and DC-only wells were included as controls. Where indicated, 50 µg/ml polyinosinic:polycytidylic acid (Poly I:C; Sigma-Aldrich), a synthetic double-stranded RNA analogue, was added to cultures. For blocking studies, hypoxic PTEC or CD1c^+^ DC were pre-incubated with inhibitors prior to co-culture (detailed information provided in Supplementary Methods).

Culture supernatants were harvested, and levels of soluble proteins were determined using the LEGENDplex™ Human Inflammation Panel 1 multiplex bead array (BioLegend, San Diego, CA, USA). LEGENDplex™ data were analysed using LEGENDplex^TM^ QOGNIT (v2020.12.15, San Carlos, CA, USA), with a standard curve calculated using a five-parameter logistic (5PL) regression. For heatmap generation, LEGENDplex^TM^ data were collated, and z-scores were calculated across cytokines in the dataset [78]. In selected experiments, levels of IL-1β in culture supernatants were assayed using the Human IL-1β ELISA MAX^TM^ Deluxe Set (BioLegend), following the manufacturer’s recommendations. Non-adherent cells (i.e., CD1c^+^ DC) from normoxic and hypoxic co-cultures were collected for quantitative real-time polymerase chain reaction (qRT-PCR) analysis (detailed information provided in Supplementary Methods). The cellular source of IL-1β in PTEC-CD1c^+^ DC hypoxic co-cultures was examined by intracellular staining and flow cytometric analysis (detailed information provided in Supplementary Methods).

### IHC staining

Frozen 7-µm sections of kidney tissue were fixed with 75% acetone:25% ethanol, followed by blocking of endogenous peroxidase activity and background protein. Serial sections were probed with antibodies against AQP-1 (Cat. No. sc-20810; Santa Cruz Biotechnology, Dallas, TX, USA), GPX4 (Cat. No. ab41787; Abcam, Cambridge, MA, USA), 4-HNE (Cat. No. ab46544; Abcam) or CD1c (Cat. No. AF5910; R&D Systems, Minneapolis, MN, USA). Sections were developed by applying the host-species appropriate horseradish peroxidase (HRP) polymer (Biocare Medical, Pacheco, CA, USA), followed by ImmPACT DAB peroxidase substrate (Vector Laboratories, Burlingame, CA, USA). Images were obtained using an Aperio AT Turbo (Leica Biosystems, Mt Waverley, VIC, Australia) bright field microscope. Quantitative-IHC analysis (positive pixel intensity/µm^2^ area) was undertaken from four randomly selected areas for each tissue sample using ImageScope (v12.2.2.5015, Leica Biosystems) (detailed information provided in Supplementary Methods).

### Immunofluorescence staining of ASC specks

Frozen 7-µm tissue sections were fixed with 75% acetone:25% ethanol, followed by a protein block. Sections were subsequently probed with primary antibodies against CD1c (Cat. No. AF5910; R&D Systems), ASC (Cat. No. AG-25B-0006-C100; Adipogen Life Sciences, San Diego, CA, USA) and AQP-1 (Cat. No. sc-25287; Santa Cruz), followed by fluorescent detection with Alexa Fluor™ Plus-conjugated secondary antibodies (all from ThermoFisher, Waltham, MA, USA). Slides were coverslipped in fluorescence mounting medium (Agilent Technologies, Santa Clara, CA, USA) and visualised using a Zeiss 780 NLO confocal microscope (Carl Zeiss, Hamburg, Germany). Quantitative image analysis of control/non-fibrotic (*n* = 4) and fibrotic (*n* = 4) kidney tissue was performed using QuPath (v0.3.2, University of Edinburgh, United Kingdom) [79], with detailed information in Supplementary Methods.

### Statistical analysis

Sample sizes were selected based on previous publications from our laboratory with similar experimental design [80, 81]. Data were normalised as a fold change relative to control conditions (i.e., normoxia) for each donor. All statistical tests were performed using GraphPad Prism (v9.0.1; GraphPad Software, La Jolla, CA, USA). Statistical comparisons between unpaired groups were performed using a Welch’s *t* test. Comparisons between paired groups were performed using a Student’s *t* test and multiple paired comparisons were performed using a one-way analysis of variance (ANOVA) with Tukey’s multiple-comparison post hoc test. *P* values ≤0.05 were considered statistically significant.

## Supplementary information

Supplementary Material

Supplementary Material - Full-length Western blots

Reproducibility Checklist

## Data Availability

The datasets generated and/or analysed during this study are available from the corresponding author on reasonable request.
