# Supplementary material for: Hypoxic human proximal tubular epithelial cells undergo ferroptosis and elicit an NLRP3 inflammasome response in CD1c+ dendritic cells
Source: Cell Death Dis. 2022 Aug 27;13(8):739. doi: 10.1038/s41419-022-05191-z (PMC9420140; doi:10.1038/s41419-022-05191-z)
Supplement: Supplementary file 1 — Supplementary Material [file 41419_2022_5191_MOESM1_ESM.docx]

**SUPPLEMENTARY METHODS**

## HMGB1 Immunoassay

PTEC cell culture supernatants were collected at 72 hours and HMGB1 levels analyzed using the human HMGB1 enzyme-linked immunosorbent assay (ELISA) kit (Aviva Systems Biology, San Diego, CA) as per the manufacturer's instructions.

## Mitochondrial superoxide detection

Mitochondrial superoxide production was evaluated using MitoSOX™ Red (Invitrogen, Grand Island, NY, USA). Briefly, harvested PTEC were incubated with MitoSOX reagent (1µM, 37°C, 30 min), with mitochondrial superoxide production (expressed as MitoSOX^+^ cells) determined by flow cytometry.

## Assessment of mitochondrial membrane potential (ΔΨ_mt_)

Mitochondrial membrane potential (ΔΨ_mt_) assessments were performed using cationic dye JC-1 (Invitrogen). Briefly, PTEC were treated for 24 hours under normoxic or hypoxic conditions. Cultured PTEC were harvested and incubated with JC-1 (10µl of 200µM stock, 37°C, 30 min), with JC-1 red fluorescence emission (~590nm) and green fluorescence emission (~529nm) detected by flow cytometry performed on a LSR Fortessa (BD Biosciences, San Jose, CA, USA). The delta median fluorescence intensity (ΔMFI; MFI test – MFI unstained control) for JC-1 red and green fluorescence were calculated, with the red/green fluorescence ratio representing the ΔΨ_mt_ for each sample.

## Annexin-V/Propidium Iodide (PI) necrosis assay

The Annexin-V Detection kit I (BD Biosciences) was used to assess PTEC necrosis. Briefly, harvested PTEC were incubated with Annexin-V FITC and PI in binding buffer for 15 min at room temperature. The percentage of Annexin-V^+^ PI^+^ necrotic cells was determined by flow cytometry performed on a LSR Fortessa (BD Biosciences).

## Protein expression by Western blotting

PTEC were lysed with urea lysis buffer (8M urea, 1% SDS, 100mM NaCl, 10mM Tris (pH 7.5); all reagents from Sigma-Aldrich, St Louis, MO, USA) and protein concentration determined using the Pierce^TM^ bicinchoninic acid (BCA) protein assay kit (Thermo Fisher Scientific, Waltham, MA, USA). Polyacrylamide gel electrophoresis (PAGE) was undertaken using standard reagents from Thermo Fisher Scientific. Samples were denatured for 5 min at 95°C, loaded onto Bolt™ 4-12% Bis-Tris Plus Gels, run at 165V for 38 min and transferred to a nitrocellulose membrane at 10V for 68 min. Membranes were blocked for 1 hour at room temperature with Intercept (PBS) blocking buffer (LI-COR, Lincoln, NE, USA) and subsequently probed with primary antibodies (Ab) overnight at 4°C, including γ-H2AX (Ser139) (1:750; Mouse monoclonal IgG; Clone JBW301; Cat. No. 05-636-I; Merck-Millipore, Burlington, MA, USA), cleaved caspase-3 (Asp175) (1:500; Rabbit polyclonal; Cat. No. 9661; Cell Signaling Technology, Danvers, MA, USA), peptidyl-prolyl isomerase F (PPIF) (1:500; Rabbit polyclonal IgG; Cat. No. SAB4500035; Sigma-Aldrich), mixed lineage kinase domain-like protein (MLKL) (phospho S358) (1:250; Rabbit monoclonal IgG; Clone EPR9514; Cat. No. ab187091; Abcam, Cambridge, MA, USA), glutathione peroxidase 4 (GPX4) (1:750; Rabbit polyclonal IgG; Cat. No. ab41787; Abcam), β-tubulin (1:2000; Rabbit polyclonal IgG; Cat No. ab6046; Abcam) and β-actin (1:1000; Mouse monoclonal IgG; Clone mAbcam 8224; Cat. No. ab8224; Abcam). Proteins were visualized with IRDye 800CW goat anti-mouse (1:15,000; Millennium Science, Mulgrave, VIC, Australia) or IRDye 680LT goat anti-rabbit (1:20,000; Millennium Science) using the Odyssey CLX (LI-COR). Quantitative analysis of protein intensities relative to β-tubulin or β-actin loading controls was performed using Image Studio 5.2 software (LI-COR).

## Cell viability measurements of human primary PTEC

Cell viability was investigated using the colorimetric MTT (3-(4,5-dimethylthiazol-2-yl)-2,5-diphenyltetrazolium bromide) Cell Proliferation Assay kit (Molecular Probes, Eugene, OR, USA). PTEC were seeded (20,000 cells/well in DM) in triplicate in 96-well flat-bottom plates to allow overnight adherence and then cultured (without prior irradiation) for a further 48 hours in fresh, conditioned DM under normoxic or hypoxic conditions. MTT solution (10µl of 12mM stock) was administered to PTEC, followed by a 2.5 hour incubation at 37°C. The MTT-containing medium was subsequently removed and DMSO applied to the cells, followed by a 10 min incubation at 37°C. Absorbance values at 540nm were determined using a Powerwave X52 microplate reader (BioTek Instruments, Winooski, VT, USA).

## 4-Hydroxynonenal (4-HNE) immunofluorescence staining of human primary PTEC

PTEC were seeded (100,000 cells/well in DM) onto sterile coverslips in 24-well flat-bottom plates to 70-80% confluence. PTEC were irradiated with 30 Gy and then cultured for 72 hours in fresh, conditioned DM under normoxic or hypoxic conditions. Following the treatment period, the cells were fixed with 4% paraformaldehyde (Sigma-Aldrich) in phosphate-buffered saline (PBS; Life Technologies, Grand Island, NY, USA) at room temperature for 10 min, followed by permeabilization with 0.5% Triton X-100 (Sigma-Aldrich) at room temperature for 30 min and a protein block with 3% bovine serum albumin (Sigma-Aldrich) in PBS at room temperature for 60 min. PTEC were probed with primary antibodies against 4-HNE (1:100; Goat polyclonal IgG; Cat. No. ab46544; Abcam) and β-tubulin (1:1000; Rabbit polyclonal IgG; ab6046; Abcam) at 4°C overnight in a humidified chamber. Coverslips were washed thrice with PBS and secondary antibodies applied. Fluorescent detection was obtained by secondary incubation with Alexa Fluor-488 anti-goat IgG and Alexa Fluor-555 anti-rabbit IgG (1:500; all from Life Technologies) at 37°C for 30 min under humid conditions. Nuclei were stained with DAPI (1:10,000, Invitrogen). Coverslips were mounted on slides in fluorescence mounting medium (Dako Omnis; Agilent Technologies, Santa Clara, CA, USA). Slides were imaged using a DeltaVision deconvolution microscope (GE Healthcare, Pittsburgh, PA, USA), with image processing and analysis using ImageJ software (NIH, Bethesda, MD, USA).

To quantify cellular 4-HNE, an outline was drawn around each cell using the β-tubulin channel, with area and integrated density of 4-HNE fluorescence for each cell measured. In addition, calculations for background mean fluorescence (3-4 randomly selected areas per image) were recorded. The corrected total cellular fluorescence (CTCF) for each cell was then calculated using the formula: CTCF = integrated density of selected cell – (area of selected cell x mean fluorescence of background readings).^S1^ The mean CTCF of >60 cells per experimental condition was then used for comparisons between samples.

**Image-iT^TM^ Lipid Peroxidation assay**

PTEC were seeded (30,000 cells/well in DM) into 96-well, black/clear bottom plates to 70-80% confluence. PTEC were irradiated with 30 Gy and then cultured for 48 hours in fresh, conditioned DM under normoxic or hypoxic conditions. Following the treatment period, the cells were incubated with 10μM of BODIPY 581/591 C11 reagent (Image-iT^TM^ lipid peroxidation kit, ThermoFisher Scientific) at 37°C for 30 minutes. Wells were washed three times with pre-warmed PBS, before 200μL Live Cell Imaging Solution (ThermoFisher Scientific) was applied to each well. The plates were visualized on a Zeiss 780 NLO confocal microscope (Carl Zeiss, Hamburg, Germany). Tile-scan images of both FITC and Texas Red^®^ channels were captured from each well and median channel intensity was quantified using QuPath (v0.3.2, University of Edinburgh, United Kingdom).^S3^ The ratio of oxidized 510 nm (green)/reduced 590 nm (red) fluorescence representing lipid peroxidation was determined for each well, with the fold change of the oxidized/reduced ratio relative to normoxia calculated for each PTEC donor.

## Human CD1c^+^ DC Isolation

Leukocyte-rich buffy coats were obtained from healthy blood donors (Australian Red Cross Lifeblood, Kelvin Grove, Queensland, Australia). Mononuclear cells were isolated using SepMate™ isolation tubes (Stemcell Technologies, Vancouver, Canada) and Ficoll Paque™ Plus density gradient centrifugation (Cytiva Lifesciences, Marlborough, MA, USA). CD1c^+^ DC were then purified from mononuclear cells using the Human CD1c (BDCA-1) ^+^ Dendritic Cell Isolation kit (Mitenyi Biotec, Bergisch Gladbach, Germany) according to the manufacturer's instructions [purity of >90% lineage (CD3, CD14, CD19, CD20, CD56)^-^ human leukocyte antigen (HLA)-DR^+^ cells; Supplementary Figure S1].

For selected experiments (i.e. quantitative RT-PCR assays), CD1c^+^ DC were further purified by labelling the magnetic bead-enriched positive fraction with LIVE/DEAD^®^ Fixable Near-IR Dead Cell reagent (to exclude dead cells), Brilliant Violet 421-conjugated CD1c (Cat. No. 331526; Biolegend, San Diego, CA, USA) and a PE-conjugated mouse anti-human lineage cocktail of CD3 (Cat. No. 300308; Biolegend), CD14 (Cat. No. 555398; BD Biosciences), CD19 (Cat. No. 555413; BD Biosciences), CD20 (Cat. No. 302306; Biolegend) and CD56 (Cat. No. 555516; BD Biosciences). Flow cytometry sorting of live, lineage-PE^-^, CD1c-Brilliant Violet 421^+^ events was performed using a FACSAria™ III cell sorter (BD Biosciences). These procedures routinely yielded CD1c^+^ DC preparations of >99% purity.

## PTEC-CD1c^+^ DC Co-Cultures

PTEC for co-culture experiments were seeded in DM in 96-well flat-bottom plates and grown to 70-80% confluence, irradiated with 30 Gy and then cultured for 72 hours in 200µl fresh, conditioned DM under normoxic or hypoxic conditions.

Human CD1c^+^ DC were resuspended at 1.33 × 10^6^ cells/ml in Complete Medium (CM), consisting of RPMI 1640, supplemented with 36.7% heat-inactivated fetal bovine serum (FBS), 100U/ml penicillin, 100mg/ml streptomycin, 2mM L-glutamine, 1mM sodium pyruvate, 0.1mM non-essential amino acids, 10 mM HEPES buffer solution (all from Invitrogen) and 50µM 2-mercaptoethanol (Sigma-Aldrich). CD1c^+^ DC (100,000 cells in CM; 75µl total volume) were added to the pre-conditioned PTEC (without removal of PTEC DM culture medium) and co-cultured for 24 hours (unless otherwise specified) under normoxic or hypoxic conditions (10% FBS final concentration; 275µL final volume). PTEC and DC only wells were included as controls. Where indicated, 50µg/ml polyinosinic:polycytidylic acid (Poly I:C; Sigma–Aldrich), a synthetic double-stranded RNA analogue, was added to cultures.

For inhibition studies, CD1c^+^ DC were pre-incubated under hypoxic conditions at 37°C for 30 min (prior to co-culture with PTEC) with caspase-1/4 inhibitor VX-765 (final concentration in co-culture of 20µM; MedChemExpress, Monmouth Junction, NJ, USA) or NLRP3 inflammasome inhibitor MCC950 (final concentration in co-culture of 50µM; Sigma-Aldrich). For inhibition of PTEC ferroptosis, 10µM ferrostatin-1 (Sigma-Aldrich) was added to hypoxic PTEC for the 72 hour treatment period prior to co-culture with CD1c^+^ DC. PTEC-derived ATP was neutralized by pre-incubating hypoxic PTEC for 30 min (prior to co-culture with CD1c^+^ DC) with apyrase (final concentration in co-culture of 1U/ml; Sigma-Aldrich).^S2^ PTEC-derived HMGB1 was neutralized by pre-incubating hypoxic PTEC for 120 min (prior to co-culture with CD1c^+^ DC) with Ultra-low endotoxin, azide-free (Ultra-LEAF™) anti-human HMGB-1 (final concentration in co-culture of 5µg/ml; Mouse monoclonal IgG2b; Clone 3E8; Cat. No. 651413; Biolegend) or Ultra-LEAF IgG2b isotype control antibody (final concentration in co-culture of 5µg/ml; Mouse monoclonal IgG2b; Clone MG2b-57; Cat. No. 401215; Biolegend).

## Quantitative real-time polymerase chain reaction (qRT-PCR) analysis of CD1c^+^ DC from normoxic and hypoxic co-cultures

Non-adherent cells (i.e. CD1c^+^ DC) from normoxic and hypoxic co-cultures were aspirated into Eppendorf tubes for RNA isolation. Total RNA was isolated from cells using the RNeasy Micro Kit with on-column DNase digestion (QIAGEN, Hilden, Germany) according to the manufacturer’s instructions. Complementary DNA (cDNA) synthesis was performed using the iScript™ Advanced cDNA synthesis kit (Bio-Rad Laboratories, Gladesville, New South Wales, Australia).

qRT-PCR for *NLPR3* and β-2-microglobulin (*B2M*) were performed using PrimePCR^TM^ primers (NLRP3 – Unique Assay ID qHsaCID0036694; B2M – qHsaCID0015347; Bio-Rad Laboratories) and SsoAdvanced™ Universal SYBR^®^ Green Supermix (Bio-Rad Laboratories). *B2M* was used for normalization of cDNA input, and qRT-PCR performed using a CFX96 Touch Real-Time PCR Detection system (Bio-Rad) according to the manufacturer’s instructions: initial denaturation at 95°C for 2 min, followed by 45 cycles at 95°C for 5 s and 60°C for 30 s; amplification specificity was confirmed by melting curve analysis. Data analysis was performed using the Bio-Rad CFX Manager Tool (v3.0.1224.1015) and data were normalised using the delta delta C_t_ (ΔΔC_t_) method.

## Flow cytometric analysis of intracellular IL-1β expression in hypoxic co-cultures

For analysis of intracellular IL-1β expression, protein transport inhibitor Brefeldin A (final concentration in co-culture of 5µg/ml; Biolegend) or caspase-1/4 inhibitor VX-765 (final concentration in co-culture of 20µM; MedChemExpress) were added to PTEC-CD1c^+^ DC hypoxic co-cultures at the beginning of the incubation period. Following 4 hours of co-culture,

cells were harvested and labelled with LIVE/DEAD^®^ Fixable Near-IR Dead Cell reagent (to exclude dead cells), CD45-Brilliant Violet 510 (Cat. No. 304036; Biolegend) (to differentiate PTEC and DC), and lineage (CD3, CD14, CD19, CD20, CD56)-PE, HLA-DR-Brilliant Ultra Violet 737 (Cat. No. 748339; BD Biosciences) and CD1c-Brilliant Violet 421 (Biolegend) antibodies (to identify CD1c^+^ DC). Cells were fixed and permeabilized using the Fixation/Permeabilization Solution Kit (BD Biosciences) and stained with IL-1β-FITC (Cat. No. 340515; BD Biosciences) for flow cytometric analysis.

## Immunohistochemistry (IHC) staining

Frozen 7µm sections of kidney tissue were fixed with 75% acetone: 25% ethanol for 5 min at room temperature. Endogenous peroxidase activity was blocked in serial sections with 1% H_2_O_2_ for 10 min, followed by a protein block with Background Sniper Blocking Reagent (Biocare Medical, Pacheco, CA, USA) for 30 min at room temperature for Aquaporin-1 (AQP-1) and GPX4, or 2% Bovine Serum Albumin (BSA; Sigma-Aldrich) for 30 min at room temperature for 4-hydroxynonenal (4-HNE) and CD1c. Serial sections were probed with anti-AQP-1 (1:500; Rabbit polyclonal IgG; Cat. No. sc-20810; Santa Cruz Biotechnology, Dallas, TX, USA), anti-GPX4 (1:200; Rabbit polyclonal IgG; Cat. No. ab41787; Abcam), anti-4-HNE (1:1200; Goat polyclonal IgG; Cat. No. ab46544; Abcam) or anti-CD1c (1:500; Goat polyclonal IgG; Cat. No. AF5910; R&D Systems, Minneapolis, MN, USA) for 1 hour at room temperature. AQP-1 and GPX4 tissue sections were washed and a rabbit horse radish peroxidase (HRP) polymer (Biocare Medical) was applied for 30 min at room temperature. 4-HNE and CD1c tissue sections were washed, with a goat HRP polymer system (Biocare Medical) applied according to the manufacturer's instructions. Peroxidase activity was developed in all sections with ImmPACT DAB peroxidase substrate (Vector Laboratories, Burlingame, CA, USA) for 5 min. Sections were lightly counterstained with hematoxylin and mounted using DPX Mounting Media. Images were obtained using an Aperio AT Turbo (Leica Biosystems, Mt Waverley, VIC, Australia) bright field microscope. Quantitative-IHC analysis (positive pixel intensity/µm^2^ area) was undertaken from four randomly selected areas for each tissue sample using ImageScope (v12.2.2.5015, Leica Biosystems).

**Immunofluorescence staining of ASC specks**

Frozen 7µm tissue sections were fixed with 75% acetone: 25% ethanol for 5 min at room temperature, followed by a protein block with 10% Donkey Serum for 30 min at room temperature. Sections were subsequently probed with primary antibodies against **CD1c (1:100; Goat polyclonal IgG; Cat. No. AF5910; R&D Systems), ASC** (1:200; Rabbit polyclonal IgG; Cat. No. AG-25B-0006-C100; Adipogen Life Sciences, San Diego, CA, USA) and AQP-1 (1:50; Monoclonal mouse IgG; Clone B-11; Cat. No. sc-25287; **Santa Cruz)** overnight at 4°C in a humidified chamber. Fluorescent detection was obtained by secondary incubation with Alexa Fluor™ Plus 488 anti-goat IgG, Alexa Fluor™ Plus 555 anti-rabbit IgG and Alexa Fluor™ Plus 647 anti-mouse IgG (1:500 dilutions; all from Thermo Fisher) for 30 min at room temperature. Nuclei were stained with DAPI (1:10,000; Sigma-Aldrich). Slides were coverslipped in fluorescence mounting medium (Agilent Technologies) and visualized using a Zeiss 780 NLO confocal microscope (Carl Zeiss).

Quantitative image analysis of control/non-fibrotic (n=4) and fibrotic (n=4) kidney tissue was performed using QuPath (v0.3.2, University of Edinburgh, United Kingdom).^S3^ Briefly, CD1c^+^, ASC speck^+^ and AQP-1^+^ cells were enumerated in five randomly selected areas for each tissue sample. Each area was assessed using the Watershed Cell Detection algorithm,^S3,4^ with staining intensity threshold-based cell classification identifying CD1c^+^ cells or AQP1^+^ cells, followed by a subcellular spot detection to identify nucleated cells with positive staining for ASC. The final count presented is the mean value (mean cells/mm^2^) from the five randomly selected areas for each tissue sample.

**SUPPLEMENTARY FIGURES**


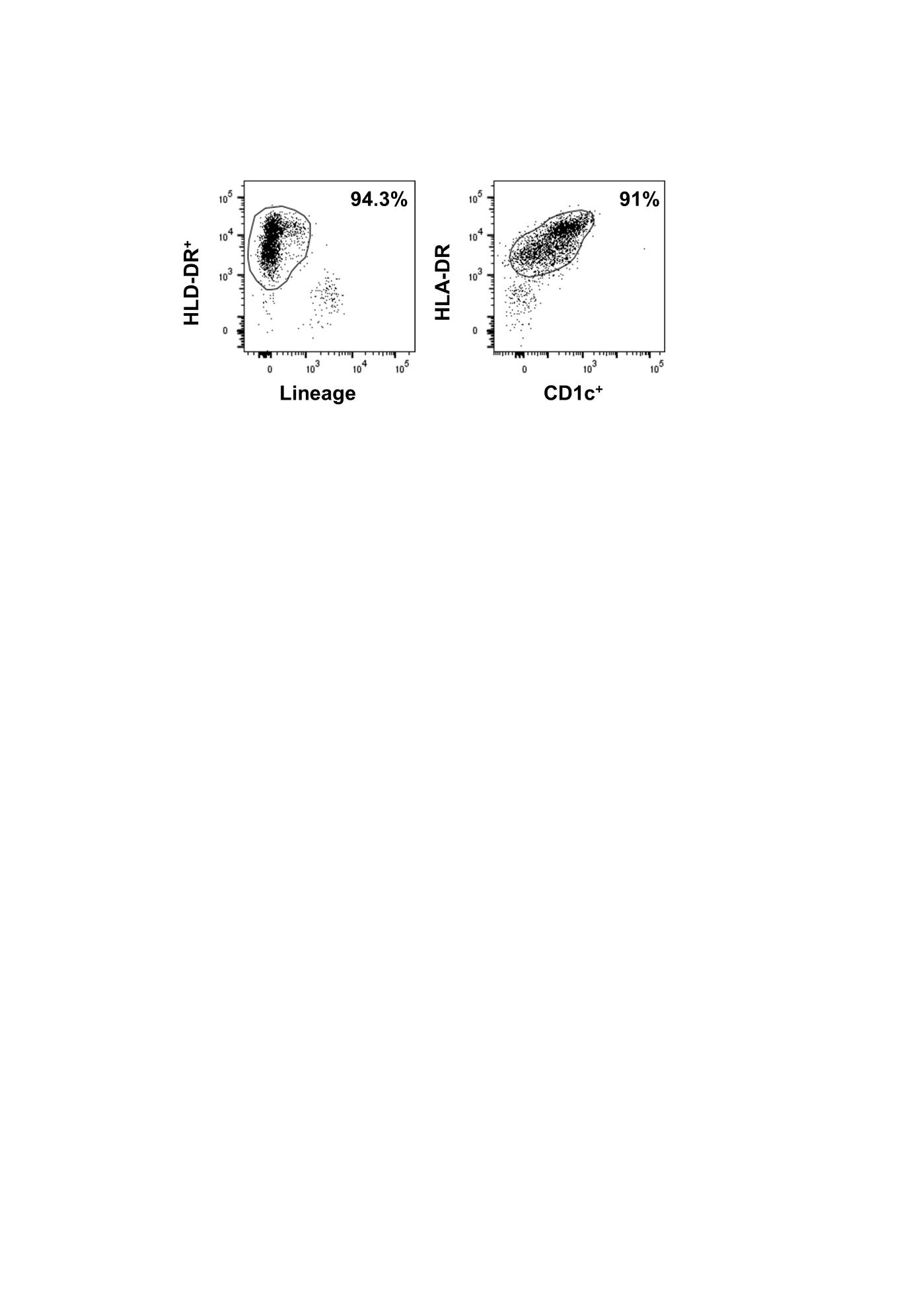


**Supplementary Figure S1.** Magnetic-bead purification of human CD1c^+^ DC. Gated on live single cells, the first plot represents the CD1c^+^ DC-enriched fraction defined as lineage^-^ HLA-DR^+^ cells and the second plot shows the fraction defined as CD1c^+^ HLA-DR^+^ cells. The dot plots are from a single representative experiment.


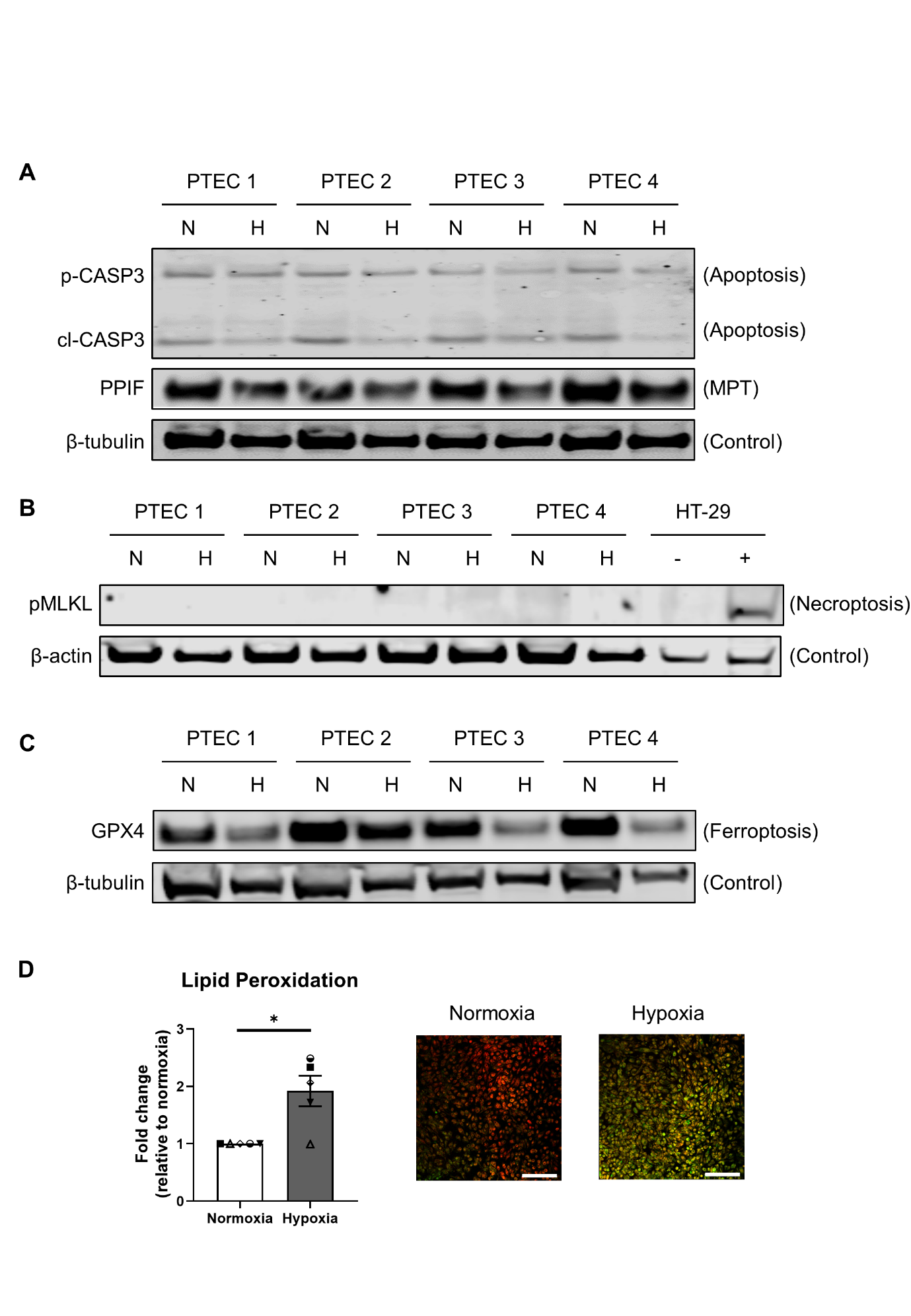


**Supplementary Figure S2. (A)** Western blot for pro-caspase-3 (p-CASP3) and cleaved caspase-3 (cl-CASP3), PPIF and loading control β-tubulin for four individual donor PTEC cultured under normoxic (N) and hypoxic (H) conditions (15µg total protein per lane). **(B)** Western blot for pMLKL (S358) and loading control β-actin for four individual donor PTEC cultured under normoxic (N) and hypoxic (H) conditions (15µg total protein per lane). HT-29 cells cultured in the absence (-) or presence (+) of z-VAD-FMK (20µM) for a total of 7.5 hrs plus LCL161 (100nM) and TNF-α (20ng/mL) for a total of 7 hrs are included as a positive control for pMLKL (necroptosis) detection. **(C)** Western blot for GPX4 and loading control β-tubulin for four individual donor PTEC cultured under normoxic (N) and hypoxic (H) conditions (15µg total protein per lane). **(D)** Left panel: Fold changes (relative to normoxia) in lipid peroxidation (measured as ratio of oxidized 510 nm (green)/reduced 590 nm (red) fluorescence) for PTEC cultured under normoxic and hypoxic conditions. Bar graphs represent mean ± SEM. Symbols represent individual donor PTEC; n=5. *p<0.05, Paired t-test. Right panel: Immunofluorescent labelling of representative PTEC cultured under normoxic and hypoxic conditions and stained with Image-iT^TM^ Lipid Peroxidation Sensor. Lipid peroxidation under hypoxic conditions is highlighted by the shift in fluorescence from red to green. Scale bars represent 200µm.


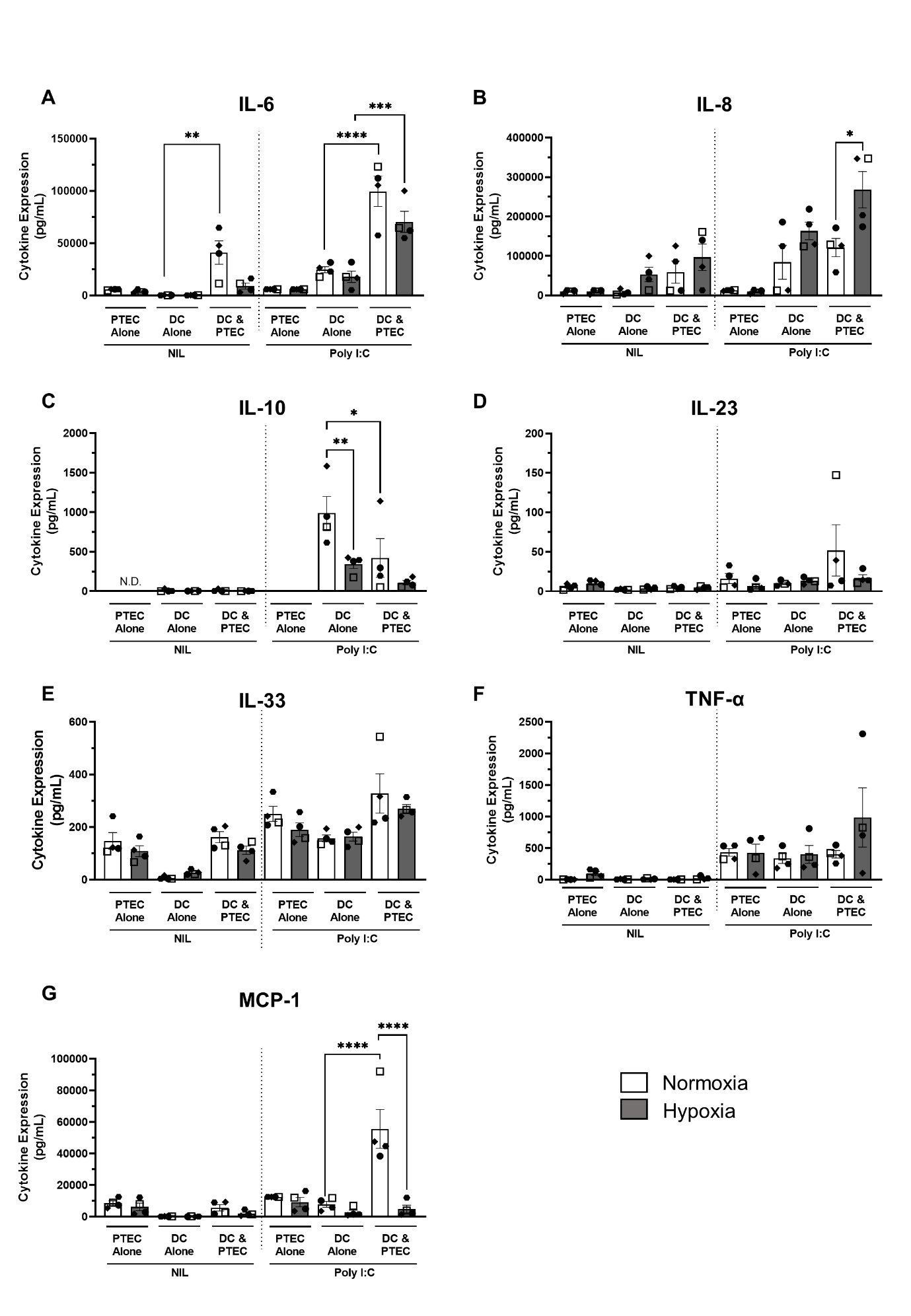


**Supplementary Figure S3.** IL-6 **(A)**, IL-8 **(B)**, IL-10 **(C)**, IL-23 **(D)**, IL-33 **(E)**, MCP-1 **(F)** and TNF-α **(G)** protein levels (measured by LEGENDplex™ assay; pg/ml) following 24 hour co-culture (DC & PTEC) of flow cytometry sorted CD1c^+^ DC with pre-conditioned normoxic (white bars) or hypoxic (grey bars) PTEC in the absence (Nil) or presence of poly I:C. PTEC alone and DC alone cultures are included as controls; N.D.- not detectable. Bar graphs represent mean ± standard error mean (SEM). Symbols represent individual donor PTEC experiments; n=4. *p<0.05, **p<0.01, ***p<0.001, ****p<0.0001, one-way ANOVA with Tukey's multiple-comparison test.

**SUPPLEMENTARY REFRERNCES**

S1. McCloy RA, Rogers S, Caldon CE, Lorca T, Castro A, Burgess A. Partial inhibition of Cdk1 in G 2 phase overrides the SAC and decouples mitotic events. *Cell Cycle* **13,** 1400-1412 (2014).

S2. DeLalio LJ, Masati E, Mendu S, Ruddiman CA, Yang Y, Johnstone SR*, et al.* Pannexin 1 channels in renin-expressing cells influence renin secretion and blood pressure homeostasis. *Kidney Int* **98,** 630-644 (2020).

S3. Bankhead P, Loughrey MB, Fernandez JA, Dombrowski Y, McArt DG, Dunne PD*, et al.* QuPath: Open source software for digital pathology image analysis. *Sci Rep* **7,** 16878 (2017).

S4. Roerdink JBTM, Meijster A. The Watershed Transform: Definitions, Algorithms and Parallelization Strategies. *Fundamenta Informaticae* **41,** 187-228 (2000).
