# Supplementary figures and images for: Hypoxic human proximal tubular epithelial cells undergo ferroptosis and elicit an NLRP3 inflammasome response in CD1c+ dendritic cells
Source: Cell Death Dis. 2022 Aug 27;13(8):739. doi: 10.1038/s41419-022-05191-z (PMC9420140; doi:10.1038/s41419-022-05191-z)

FIGURE 1C - Full-length Western Blot

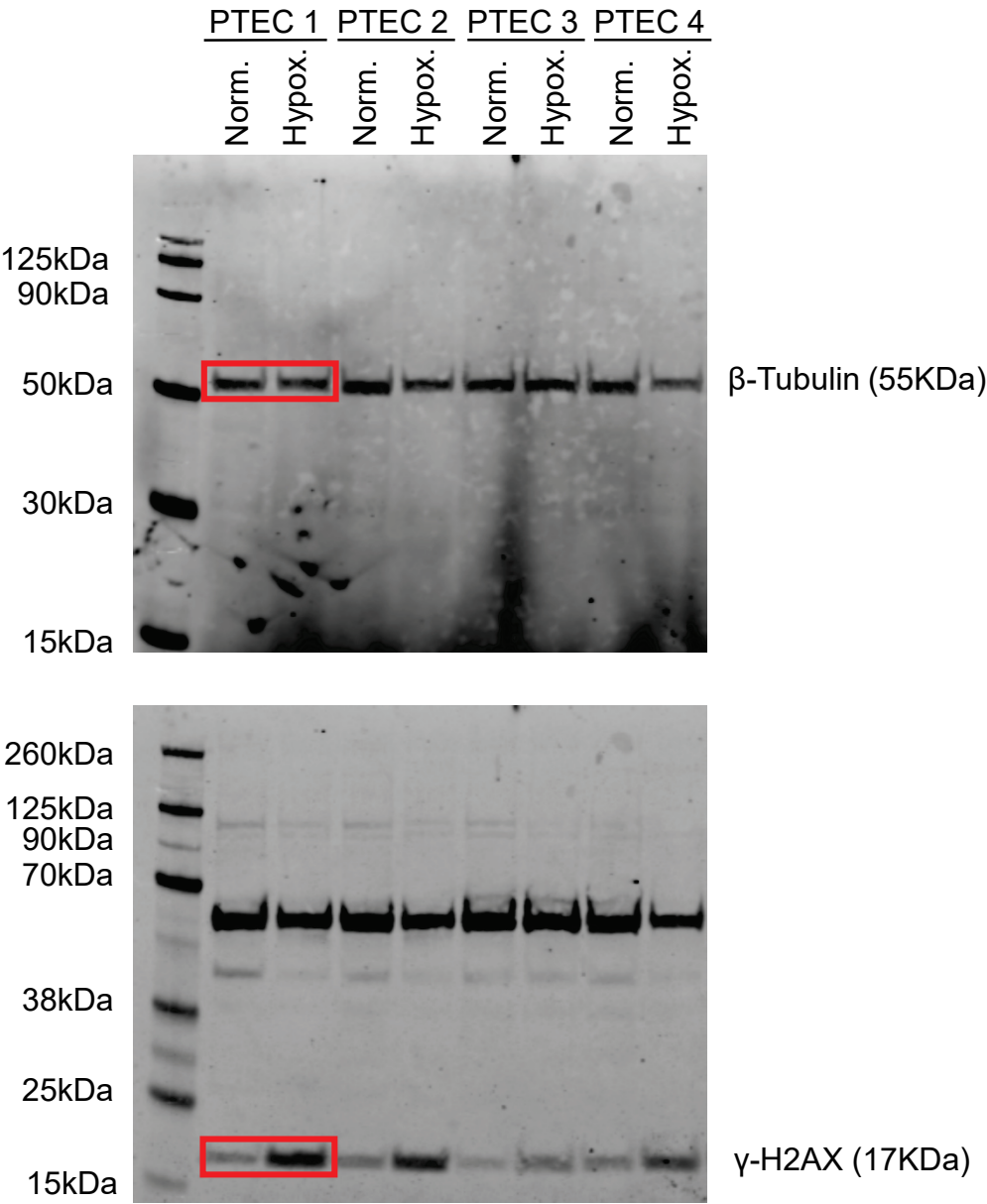

FIGURE 2B - Full-length Western Blot

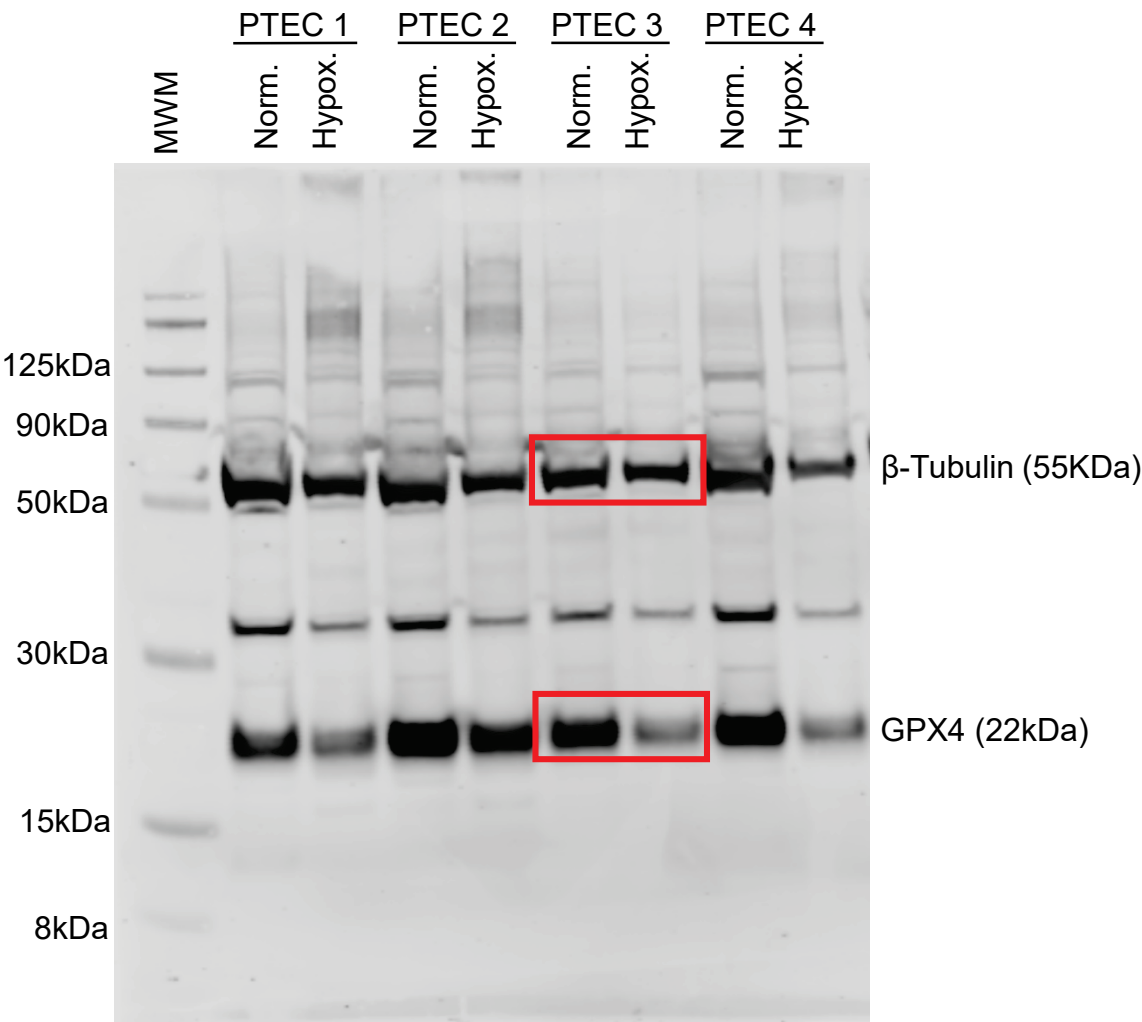

Supplement: Supplementary file 2 — Supplementary Material - Full-length Western blots [file 41419_2022_5191_MOESM2_ESM.pdf]
